# The endothelial αENaC contributes to vascular endothelial function *in vivo*

**DOI:** 10.1371/journal.pone.0185319

**Published:** 2017-09-26

**Authors:** Antoine Tarjus, Martina Maase, Pia Jeggle, Ernesto Martinez-Martinez, Céline Fassot, Laurent Loufrani, Daniel Henrion, Pernille B. L. Hansen, Kristina Kusche-Vihrog, Frederic Jaisser

**Affiliations:** 1 Inserm U1138, Centre de Recherches des Cordeliers, Paris, France; 2 Université Pierre et Marie Curie, Paris, France; 3 Institute of Physiology II, University of Muenster, Muenster, Germany; 4 UMR CNRS 6214 Inserm 1083, Université d'Angers, Angers, France; 5 Cardiovascular and Renal Research, University of Southern Denmark, Odense, Denmark; 6 AstraZeneca, Gothenburg, Sweden; 7 Inserm, Clinical Investigation Centre 1433, Vandoeuvre-lès-Nancy, France; University of Pittsburgh School of Medicine, UNITED STATES

## Abstract

The Epithelial Sodium Channel (ENaC) is a key player in renal sodium homeostasis. The expression of α β γ ENaC subunits has also been described in the endothelium and vascular smooth muscle, suggesting a role in vascular function. We recently demonstrated that endothelial ENaC is involved in aldosterone-modulated endothelial stiffness. Here we explore the functional role of the endothelial αENaC subunit in vascular function *in vivo*. Compared to littermates, mice with conditional αENaC subunit gene inactivation in the endothelium only (endo-αENaC Knock Out mice) had no difference in their physiological parameters such as systolic blood pressure or heart rate. Acute and long-term renal Na^+^ handlings were not affected, indicating that endothelial αENaC subunit is not involved in renal sodium balance. Pharmacological inhibition of ENaC with benzamil blunted acetylcholine-induced nitric oxide production in mesenteric arteries from wild type mice but not in endo-αENaC ^KO^ mice, suggesting a critical role of endothelial ENaC in agonist-induced nitric oxide production. In endo-αENaC ^KO^ mice, compensatory mechanisms occurred and steady state vascular function was not altered except for flow-mediated dilation. Our data suggest that endothelial αENaC contributes to vascular endothelial function *in vivo*.

## Introduction

The endothelium is a thin monolayer of cells that line the interior surface of entire blood vessels, forming a physical barrier between circulating blood element and underlying tissues. Endothelial cells are involved in many aspects of vascular biology, including the response to shear force, modulation of blood vessel tone and blood flow [1], thus playing an important role in blood pressure regulation. However, the way how endothelial cells are able to sense the mechanical force produced by the pulsatile circulating blood and translate it into a molecular signal remains unknown. Several actors have been suggested as endothelial mechanosensors and among them, ion channels. Ion channels expressed in endothelial cells are considered to mediate “short term” responses (in the range of seconds and minutes) to shear stress and subsequently affect cytoskeleton rearrangement and synthesis and/or release of pro and anticoagulants, growth factors and vasomotor regulators [2]. Among these ion channels, a new candidate has rose these last decades: the Epithelial Sodium Channel (ENaC). ENaC is a sodium channel member of the ENaC/Degenerin superfamily, which includes the Degenerins subunits that act as mechanosensors in *Caenorhabditis elegans* [3, 4]. ENaC has been described in the endothelium for fifteen years, but its role in cell function and in vascular biology remains largely unknown [5–8].

ENaC is expressed in the renal epithelium where it is one of the key players responsible for the fine tuning of sodium reabsorption. ENaC is composed of three subunits (αENaC, βENaC and γENaC coded, respectively, by the genes *Scnn1a*, *Scnn1b* and *Scnn1g*), forming a channel expressed at the apical membrane of the cells, thus allowing sodium entry through the renal epithelium [9–11]. Regulation of sodium homeostasis is a crucial physiological process, as it maintains renal salt and water balance, blood volume, and consequently blood pressure [12, 13]. There is now evidence that ENaC opening probability is regulated by flow-induced shear stress in oocyte and in the epithelium of cortical collecting duct [14], which highlights its implication in the detection of mechanical forces (and its potential role as a direct mechanosensor).

As in the renal collecting duct (where the epithelial cells are submitted to urine flow-induced laminar shear stress), the endothelial cells support blood flow-induced laminar shear stress, thus raising the potential implication of shear stress sensing in vascular endothelium. In cultured vascular endothelial cells, ENaC mediates amiloride-sensitive Na^+^ currents [6–8]. Interestingly, its channel activity and its opening probability have been showed to be regulated by flow-induced shear stress [7, 8]. Moreover, endothelial ENaC has been reported to be involved in mechanical properties of endothelial cell: increased endothelial ENaC expression or activity increases endothelial cell stiffness both in cultured human endothelial cells and in aorta of Liddle syndrome mouse model [15]. Among the three subunits, αENaC has been shown to be necessary and sufficient to produce an inward sodium current [11] and Jeggle *et al*. showed that the aENaC subunit by itself could alter endothelial cortical stiffness [15]. The consequences of pharmacological inhibition of vascular ENaC has been studied *ex vivo* [6, 8]. However, the role of endothelial ENaC on vascular function *in vivo* has not been analyzed so far. Since ENaC is activated by flow we investigated whether it could play a role in shear stress sensing and flow-mediated dilation *in vivo*.

To address this question, we focused our study on the role of the alpha ENaC in the endothelium by using a constitutive knockout mouse model allowing deletion of the αENaC subunit in the endothelial cells.

## Materials and methods

### Mouse models

αENaC subunit (*Scnn1a*) knockout mice, thereafter called endo-αENaC ^KO^, were obtained crossing αENaC^f/f^ floxed mice (kindly provided by Bernard Rossier, Lausanne, Switzerland [16] with transgenic mice expressing Cre recombinase under the control of Tie2 promoter on a C57BL/6 genetic background (The Jackson Laboratory, USA). αENaC ^f/f^ littermates lacking the Tie2-Cre transgene were used as controls. The sequences of the primers used for genotyping are listed in S1 Table. Animals were 3-6-month-old males housed in a climate-controlled facility with a 12-hour/12-hour light/dark cycle and provided free access to food and water. Animals were euthanized with an intraperitoneal injection of a lethal dose of ketamine/xylazine and blood was withdrawn by cardiac puncture. Organs were harvested and immediately conserved in formalin or snap-frozen in liquid nitrogen and stored at -80°C unless specified otherwise. All experiments were approved by the Charles Darwin ethics committee (C2EA– 05) of Pierre et Marie Curie University, and conducted in accordance with the European legislation for the care and use of laboratory animals.

### cDNA isolation and real-time PCR of ENaC subunits

Total RNA from whole kidney were extracted using the TRIZOL® reagent (Life Technologies Corporation, USA), according to manufacturer protocol. Aortas were extracted using RNeasy Kit (Qiagen, Germany). Reverse transcription of mRNA (500 ng) was performed using Superscript II Reverse Transcriptase KIT (Life Technologies Corporation, USA). Transcript levels of genes were analyzed by real-time PCR (fluorescence detection of SYBR Green) in an iCycler iQ (Bio-Rad, USA). For each sample, mRNA levels were normalized to the housekeeping gene, 18S (*Rn18s*). The sequences of the primers are listed in S1 Table (Eurogentec, Belgium).

### Quantum dot-based immunofluorescence of αENaC at cell surface *ex vivo*

Endothelial cells of *in situ* endothelial cells of *ex vivo* aorta preparations were fixed and stained as described elsewhere [15, 17]. Briefly, αENaC abundance on the upper cell surface (facing the medium) was detected and quantified via Quantum Dot (QD)-based immunofluorescence. Therefore, a primary polyclonal rabbit anti-αENaC antibody (Santa Cruz; 1:250), a secondary QD-labeled antibody (Qdot 655 goat anti-rabbit IgG, Invitrogen; 1:800) and DAPI (4',6-diamidino-2-phenylindole; Invitrogen) were used for staining. Negative controls were established by incubating cells solely with the secondary antibody. The specificity of the primary antibody was recently demonstrated by using M-1 cells as positive control and siαENaC-transfected EA.hy926 cells [15]. Staining was verified by epi-fluorescence microscopy (microscope: Leica DMI 6000B, Leica Microsystems; camera: CoolSNAPHQ, Photometrics, USA). The QD-based immunofluorescence was quantified by counting QD/1000 μm^2^ of cell surface using ImageJ software (National Institutes of Health, USA). Images were taken in 3 different sections of the endothelial monolayer and all images were analyzed simultaneously in order to account for any variations in cell height. Staining of all groups were performed in parallel and the QD background derived from a negative control without primary antibody was subtracted from all samples.

### Measurement of endothelial stiffness using atomic force microscope

As previously described [18], the first ~1.5 cm of aortae, from heart to diaphragm, were dissected from the mice and freed from surrounding tissue. A small patch (about 1 mm^2^) of the whole aorta was prepared and attached on Cell-Tak^®^ coated glass, a tissue adhesive (BD Biosciences, Germany), with the endothelial surface facing upwards. Prior to the experiments, the preparations were kept in minimal essential medium (MEM) (Invitrogen Corp.) with the addition of 1% MEM vitamins, penicillin G (10,000 U/mL), streptomycin (10,000 μg/mL) (Biochrome AG, Germany), 1% NEAA and 20% fetal bovine serum (PAA Clone) at 37°C and 5% CO_2_.

Mechanical stiffness of the endothelial cell cortex, a layer 50–200 nm beneath the plasma membrane, of *ex vivo* aorta preparations was determined using an atomic force microscope (AFM) nano-indentation technique [19, 20]. Stiffness measurements of living endothelial cells *ex vivo* were conducted with a scanning probe microscope (MultiMode^®^ SPM, Bruker, USA) with a feedback-controlled heating device (Bruker) or an atomic force microscope integrated into an inverted fluorescence microscope (BioScope Catalyst^TM^, Bruker; combined with DMI 6000B, Leica Microsystems, Germany). During the measurements, artery preparations were bathed in HEPES-buffered solution (standard composition in mM: 135 NaCl, 5 KCl, 1 MgCl_2_, 1 CaCl_2_, 10 HEPES (N-2-hydroxyethylpiperazine-N′-2-ethanesulfonicacid, pH 7.4). AFM stiffness measurements were performed using soft cantilevers (spring constant: <20 pN/nm; Novascan) with a polystyrene sphere as the tip (diameter: 10μm). A maximal loading force of 2 nN was applied. Obtained AFM data were collected with NanoScope softwares 5.31 and V8.10 (Bruker). Stiffness values were calculated from force-distance curves using the Protein Unfolding and Nano-Indentation Analysis Software PUNIAS 3D version 1.0 release 1.8 (http://punias.voila.net).

### Physiological parameters

Systolic blood pressure and heart rate were measured by tail cuff plethysmography in trained conscious mice (three days of training). Ten measurements per mice were taken every day between 1 p.m. and 3 p.m. during five days using a BP2000 Visitech model (Bioseb, France). The values presented are the mean values obtained during five days.

Renal function and ions excretion were assessed using metabolic cages (Tecniplast, France). Mice were allowed three days of habituation with food and water *ad libidum*. For renal function and sodium handling at basal state, urine was collected on a 24 hours period. Blood was collected from the retro-orbital cavity.

For the acute sodium load challenge, 1 mEq of sodium chloride was given by gavage in 200 μl water. Urine was collected at 4, 8 and 24 hours after gavage. For amiloride (an ENaC inhibitor), a dose of 1.45 g per gram of body weight was administrated via intra-peritoneal injection in 100 μl of water. Urine was collected at 4, 8 and 24 hours after injection. Low salt diet (0.1% NaCl, SAFE, France) was given for four days and urine was collected twice a day.

Urea and creatinine (enzymatic method) were quantified using a KONELAB (ThermoFischer Scientific, USA), sodium and potassium using a flame photometer (Instrumentation Laboratory, USA). Creatinine clearance was calculated using the equation $\frac{U}{P}\times V$ where *U* is the concentration of creatinine in urine, *P* is the concentration of creatinine in plasma, and *V* is the urine flow rate in milliliters per minute.

Aldosterone plasma levels were measured by using a radioimmunoassay Coat-a-Count (Siemens, Germany). Plasmatic levels of corticosterone were measured using EIA kit (Cayman Chemical Company, USA). Assays were realized according to manufacturer’s protocols.

### Measurement of nitric oxide in mesenteric arteries of mice

Mesenteric arteries from endo-αENaC ^KO^ and wild type mice were isolated, cut open and placed with the endothelial layer facing upwards. Low levels of nitric oxide (NO) were measured with 4-amino-5-Mehtylamino-2’,7’-Difluorofluoresceince (DAF-FM) as described previously [21]. In short, vessels were incubated 4 μM DAF-FM diacetate (Molecular Probes®) followed by wash-out. The experiments were executed at an Olympus IX71microscope and visualized by excitation at 495 nm and emission at 515 nm. Full frame images were collected and analyzed for relative changes in fluorescence intensity (ΔF = (F/F0)). Endothelial cells that were in focus during the whole experiment were analyzed.

### Western blot and antibodies

Western blot analysis was performed on proteins extracted from whole aortas obtained from endo-αENaC ^KO^ and wild type mice. Samples were homogenized immediately after collection, using 150 μl of Complete Lysis-M buffer with proteases and phosphatases inhibitors cocktail (Roche, Switzerland). Extracts were centrifuged at 14000 rpm for 10 minutes at 4°C and protein concentration in the supernatant was determined by the Pierce protein assay (Bio-Rad, USA). Thirty micrograms of total proteins were loaded on 4–15% polyacrylamide TGX gel (Bio-Rad, USA) and transferred to nitrocellulose membranes using Trans Blot turbo (Bio-Rad, USA). Primary antibodies were incubated at 4°C overnight, while secondary antibodies were incubated 1h30 at room temperature. Membranes were stripped after detection of the phosphorylated protein in order to detect total unphosphorylated proteins. Antibodies and dilutions are listed in the S2 Table.

### Vascular reactivity analysis

Vascular contractile and relaxing responses to pharmacological agents and flow-dependent tone in isolated segments of mesenteric arteries were assessed as previously described [22]. Briefly, mesenteric arteries were cannulated at both ends in a video-monitored perfusion system (LSI, Burlington, VT, USA) and bathed in a PSS of the following composition (mM): 130, NaCl; 15, NaHCO_3_; 3.7, KCl; 1.2 KH_2_PO_4_; 1.2, MgSO_4_; 11, glucose; 1.6, CaCl_2_; and 5, HEPES, pH 7.4, PO_2_ 160 mmHg, PCO_2_ 37 mmHg). Pressure was maintained at 75 mmHg by a servo-perfusion system and flow was generated by a peristaltic pump. For pharmacological agents, arterial segments reactivity was estimated using first a potassium rich solution (8.10^−2^ M). After that, endothelial function was evaluated using acetylcholine (10^−6^ M) after precontraction with phenylephrine (10^−6^ M). For flow mediated dilation, diameter changes and changes in wall thickness were measured at a set pressure of 75 mmHg and flow was increased in steps. To study the effect of pharmacological inhibition of ENaC, the mesenteric arteries of C57BL/6 were incubated 10 minutes with 10^−6^ M of benzamil. At the end of each experiment, arterial segments were perfused with a Ca^2+^-free physiological salt solution containing ethylenbis-(oxyethylenenitrolo) tetra-acetic acid (EGTA; 2.10^−3^ M), papaverin (10^−4^ M) and SNP (10^−5^ M); then passive diameter of the arteries was measured at 75 mmHg [23]. Results were expressed as the percentage of passive diameter (measured diameter/passive diameter×100). All the chemicals were purchase from Sigma-Aldrich if not specified otherwise.

### Statistical analysis

The results are reported as mean ± standard error of the mean (SEM). Data analysis was performed with GraphPad Prism (V.6.04, GraphPad Software, USA). Normality of the groups was assessed through the D'Agostino & Pearson omnibus normality test. If normality was verified, the comparison of two groups was made using a t-test. Otherwise, non-parametric Wilcoxson-Mann Whitney U test for comparison of two groups was used. For comparison of more than two groups, with two parameters, a two-way ANOVA with Bonferroni post-tests was used. *P* values <0.05 were considered statistically significant. Asterisks refer to: *P≤0.05; **P≤0.01; ***P≤0.001, ****P≤0.0001. For AFM measurements, data were calculated as median (Q_1_:1^st^ quartile, Q_3_:3^rd^ quartile) and are displayed as box plots: mean (square), median (horizontal line), 25^th^ and 75^th^ percentile (box), outlier (whiskers).

## Results

### Pharmacological ENaC inhibition affect vascular function

**Fig 1:**
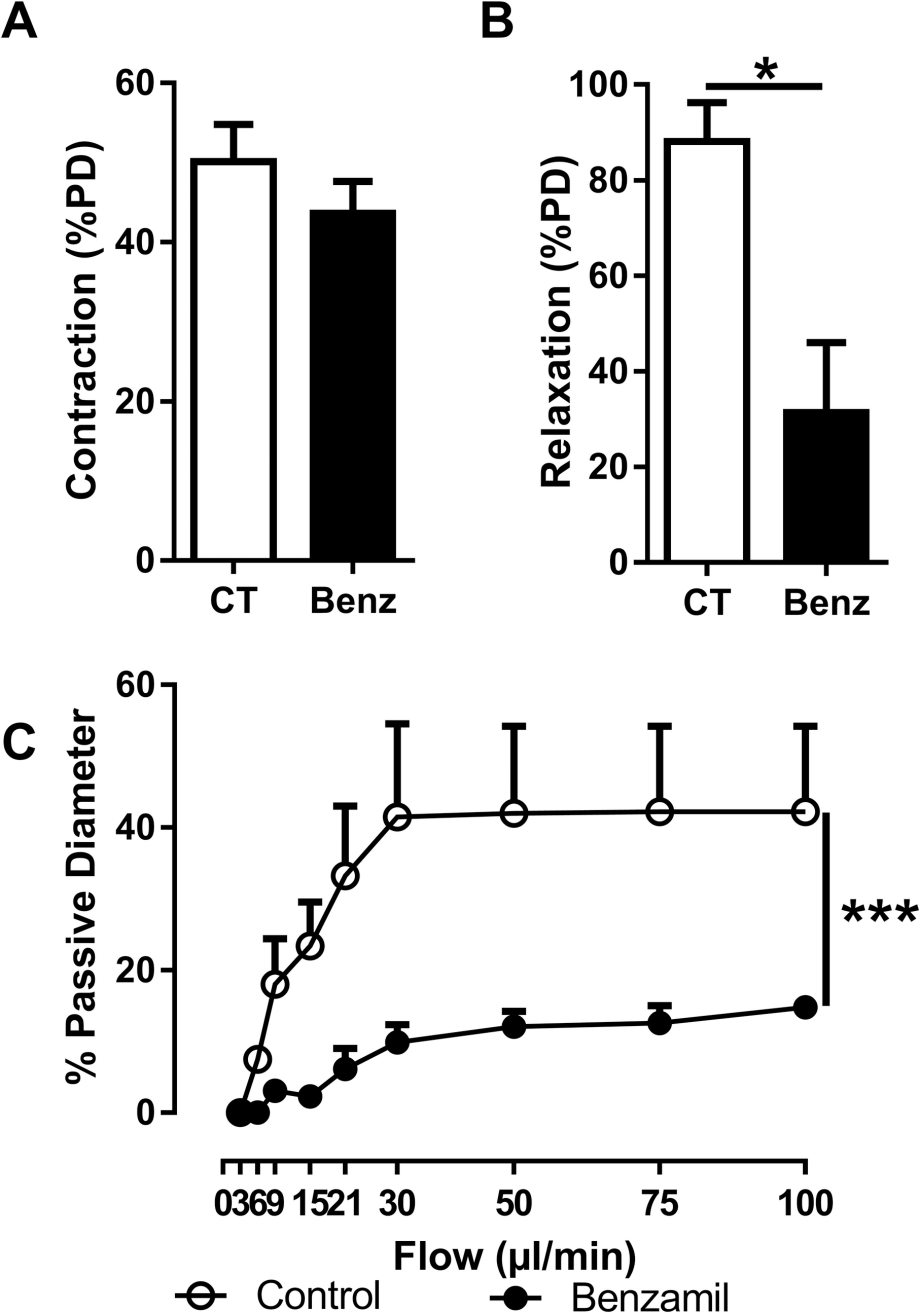
Effects of pharmacological inhibition of ENaC on vascular response mesenteric arteries. (A) Vasoconstrictive response to phenylephrine (1.10^−6^ M), an α-adrenergic agonist. Response to phenylephrine was not modified after inhibition of ENaC. (B) Endothelium-dependent dilatory response induced by acetylcholine (1.10^−6^ M). Dilation of mesenteric arteries to Ach was decreased after benzamil preincubation. (C) Flow-mediated dilation in response to increased intraluminal shear stress. Dilation to flow was drastically decreased in vessels incubated with benzamil. Values are mean ± SEM (*n* = 3–10 for each group). *P<0.05, ***P<0.001.

Pre-treatment with benzamil, a specific blocker of ENaC, had no effect on Phe-induced contraction of mesenteric arteries from C57BL/6 mice (Fig 1A). However, the endothelium-dependent dilatory response to acetylcholine was decreased by 75% (Fig 1B). By analogy with the other members of the ENaC/Degenerin superfamily, ENaC has been proposed to be a mechanotransducer and we tested whether vascular ENaC could be involved in the response to mechanical forces induced by blood flow by measuring flow-induced vasodilation in the absence or the presence of benzamil. As expected, increased intraluminal flow resulted in a large augmentation in the vessels passive diameter. Pre-incubation of the vessels with benzamil drastically blunted the flow-mediated dilation (Fig 1C) suggesting the role of endothelial ENaC subunit in shear stress signaling.

### *In vivo* αENaC deletion

**Fig 2:**
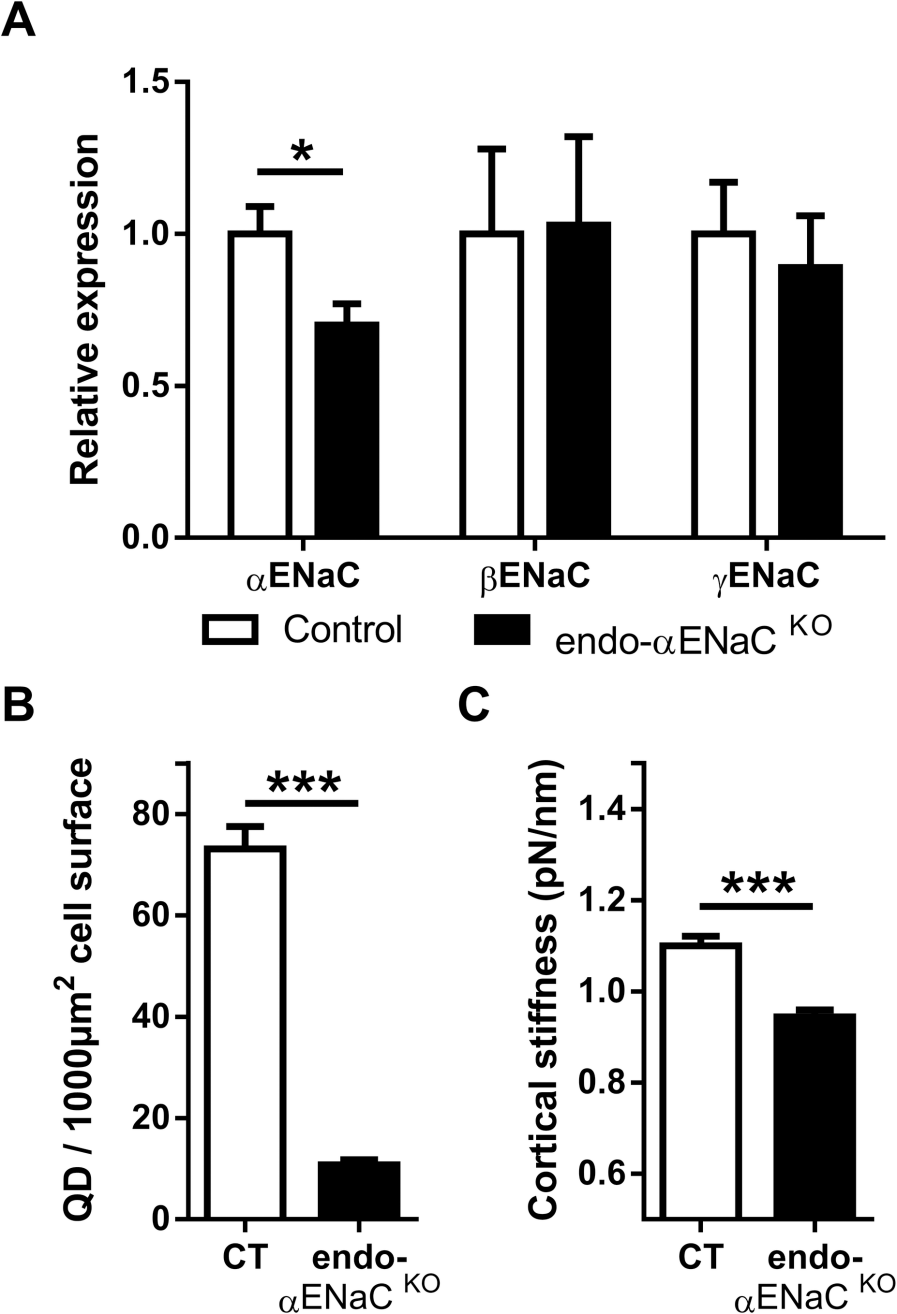
Characterization of the endo-αENaC ^KO^ mouse model. (A) Relative mRNA expression of the three ENaC subunits in the aorta of control mice (white bars) and endo-αENaC ^KO^ mice (black bars). Expression of the α subunit is decreased, but not those of the β and γ subunits. (B) Surface αENaC protein expression in aortic rings. (C) Aortic cortical stiffness of endothelial cells was determined by atomic force microscope (AFM). Values are mean ± SEM (*n* = 7–9 for each group). *P<0.05, ***P<0.001.

To determine the specific role of endothelial αENaC, we generated a mouse model with targeted endothelial αENaC subunit inactivation. These mice presented Mendelian ratio at birth. mRNA expression of the αENaC subunit was decreased by 30% in the whole aorta of endo-αENaC ^KO^ mice, as compared to control mice, while expression of the β and γENaC subunits were not modified (Fig 2A). In order to differentiate endothelial αENaC expression from non-endothelial αENaC expression, we compared mRNA expression of αENaC in aortas submitted or not to the mechanical removal of the endothelium (S1 Fig). As shown in S1A Fig, expression of αENaC was decreased in the same proportion in the control aortas without endothelium than in the KO aortas with endothelium, suggesting that most if not all of the αENaC may indeed be expressed in the endothelium. However, we cannot rule out small expression of αENaC in VSM since the removal of endothelium was not complete (as seen by the residual expression of the endothelial marker von Willebrand Factor, S1B Fig). Since Tie2 promoter is known to be expressed in non-endothelial cells [24], we also verified whether αENaC expression was altered in cells from the hematopoietic lineage. We did not see any differences between the control group and the knockout group (S2 Fig).

Apical surface expression of the αENaC subunit, as determined by quantum dot labeling, was virtually absent in the aortic endothelium of endo-αENaC ^KO^ mice, as compared to control mice (Fig 2B and S3 Fig). γENaC subunit was still present in the plasma membrane of *ex vivo* endothelial cells derived from endo-αENaC ^KO^ aorta preparations (S3 Fig). Since the endothelium function is closely related to its structure, we analyzed endothelial cell stiffness using AFM in aortic *ex vivo* preparations from endo-αENaC ^KO^ and WT mice. αENaC gene inactivation in the endothelium decreased cortical stiffness similar to benzamil treatment of aortic preparations of control mice (Fig 2C). Taken together these data indicate that αENaC gene deletion is efficient in endo-αENaC ^KO^ mice and that absence of endothelial αENaC affects endothelial stiffness in intact vessels, as previously reported in cultured endothelial cells *ex vivo* [15].

### Physiological consequences of αENaC deletion in the vascular endothelium

At basal state, systolic blood pressure and heart rate were similar between control and endo-αENaC ^KO^ mice, as well as body weight, heart/tibia length and kidney/tibia length ratio (Table 1). The structure of the vessels was not different between control and endo-αENaC ^KO^ mice (S3 Table). Creatinine clearance was similar between control and endo-αENaC ^KO^ mice (Table 1) as well as steady-state 24 h renal sodium excretion (urinary Na^+^/creatinine ratio: 10.02 ± 0.58 vs 9.43 ± 0.63). Endothelial αENaC inactivation did not affect renin RNA expression in whole kidney (renin/18S ratio: 1.00 ± 0.08 vs 1.23 ± 0.16) nor aldosterone and corticosterone plasma levels (Table 1). We next analyzed whether endothelial ENaC may affect acute renal Na^+^ handling. Endo-αENaC ^KO^ mice handled acute Na^+^ challenge similarly to control mice: acute Na^+^ load or acute ENaC inhibition with amiloride increased sodium excretion to comparable values in both groups (S4A and S4B Fig). Low salt diet decreased Na^+^ excretion with the same kinetics in endo-αENaC ^KO^ mice and control mice (S4C Fig). These results show that αENaC inactivation in vascular endothelium does not affect the classical epithelial ENaC-dependent renal Na^+^ transport.

**Table 1:** Physiological parameters of mice.

	Control (*n* = 11)			Endo-αENaC ^KO^ (*n* = 9)		
Systolic Blood Pressure (mmHg)	113	±	1	112	±	2
Heart Rate (bpm)	615	±	20	605	±	17
Body Weight (g)	29.5	±	0.9	30.0	±	1.0
Tibia Lenght (mm)	18.2	±	0.04	18.4	±	0.09
HW/TL ratio (mg/mm)	7.45	±	0.39	8.09	±	0.37
KW/TL ratio (mg/mm)	19.5	±	0.9	20.0	±	0.9
Creatinine Clearance (ml/min)	0.53	±	0.06	0.49	±	0.06
Aldosteronemia (nmol/l)	0.69	±	0.21	0.90	±	0.23
Corticosteronemia (nmol/l)	85.5	±	24.3	75.3	±	19.3

HW, Heart Weight; KW, Kidney Weight; TL, Tibia Length. Values are mean ± SEM.

### Endothelial αENaC is involved in acetylcholine-mediated endothelial nitric oxide production

**Fig 3:**
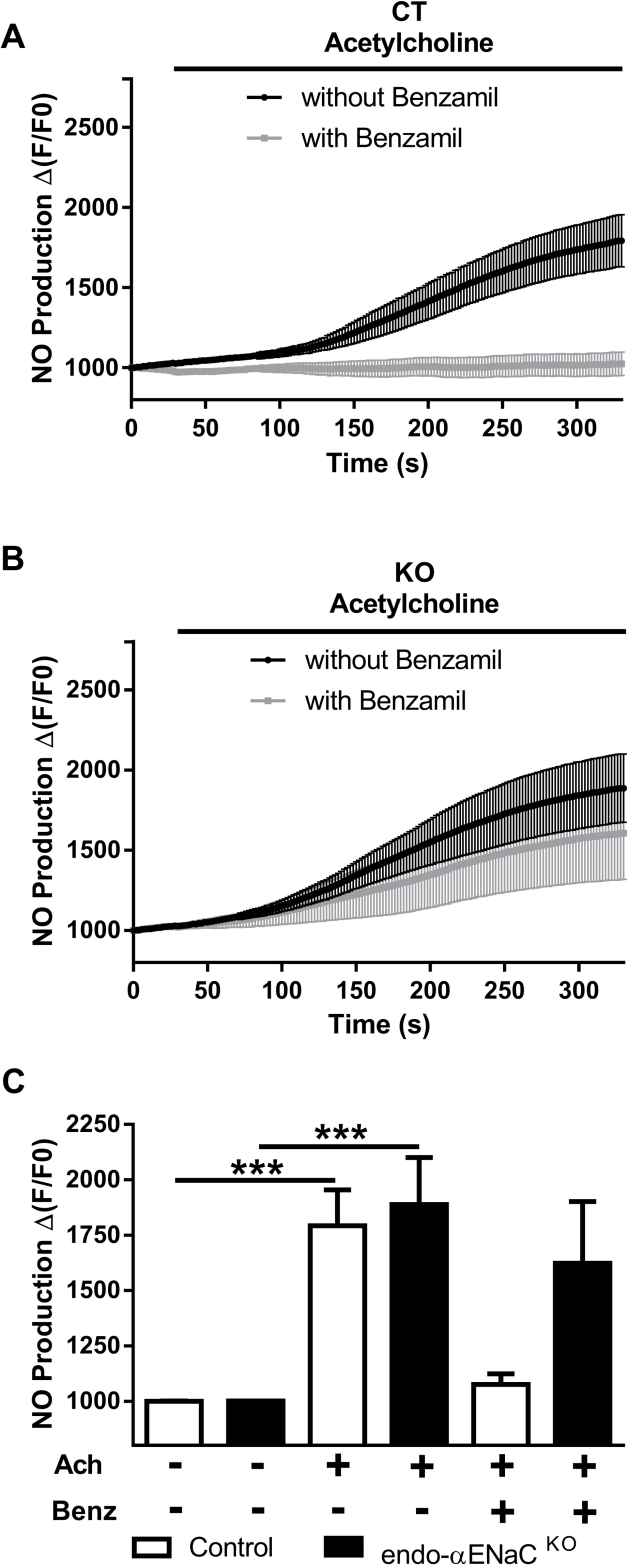
Consequences of ENaC blockade or gene inactivation on nitric oxide production upon acetylcholine stimulation. Kinetic response of NO production after stimulation with acetylcholine using fluorescent probes in mesenteric arteries of (A) control mice and (B) endo-αENaC ^KO^ mice. (C) Bar graph of NO measurements in mesenteric arteries after incubation with or without 1.10^−6^ M of acetylcholine (Ach) and 1.10^−7^ M of benzamil (Benz) in CT (white bars) and endo-αENaC ^KO^ mice (black bars). Values are mean ± SEM (*n* = 5–10 for each group). ***P<0.001.

Endothelial nitric oxide (NO) production is a hallmark of endothelial function and alteration of NO production is critical for vascular function in both physiological and pathological conditions. Agonist-mediated NO production was analyzed in isolated mesenteric arteries from control and endo-αENaC ^KO^ mice, in the presence or absence of benzamil, a pharmacological ENaC blocker. In control mice, 10^−6^ M acetylcholine induced NO release which was blocked by benzamil (Fig 3A and 3C), indicating that ENaC function contributes to physiological NO release upon acetylcholine stimulation. In endo-αENaC ^KO^ mice, acetylcholine–mediated NO release was similar than in control mice (Fig 3B and 3C) but the inhibitory effect of benzamil was lost (Fig 3B and 3C).

**Fig 4:**
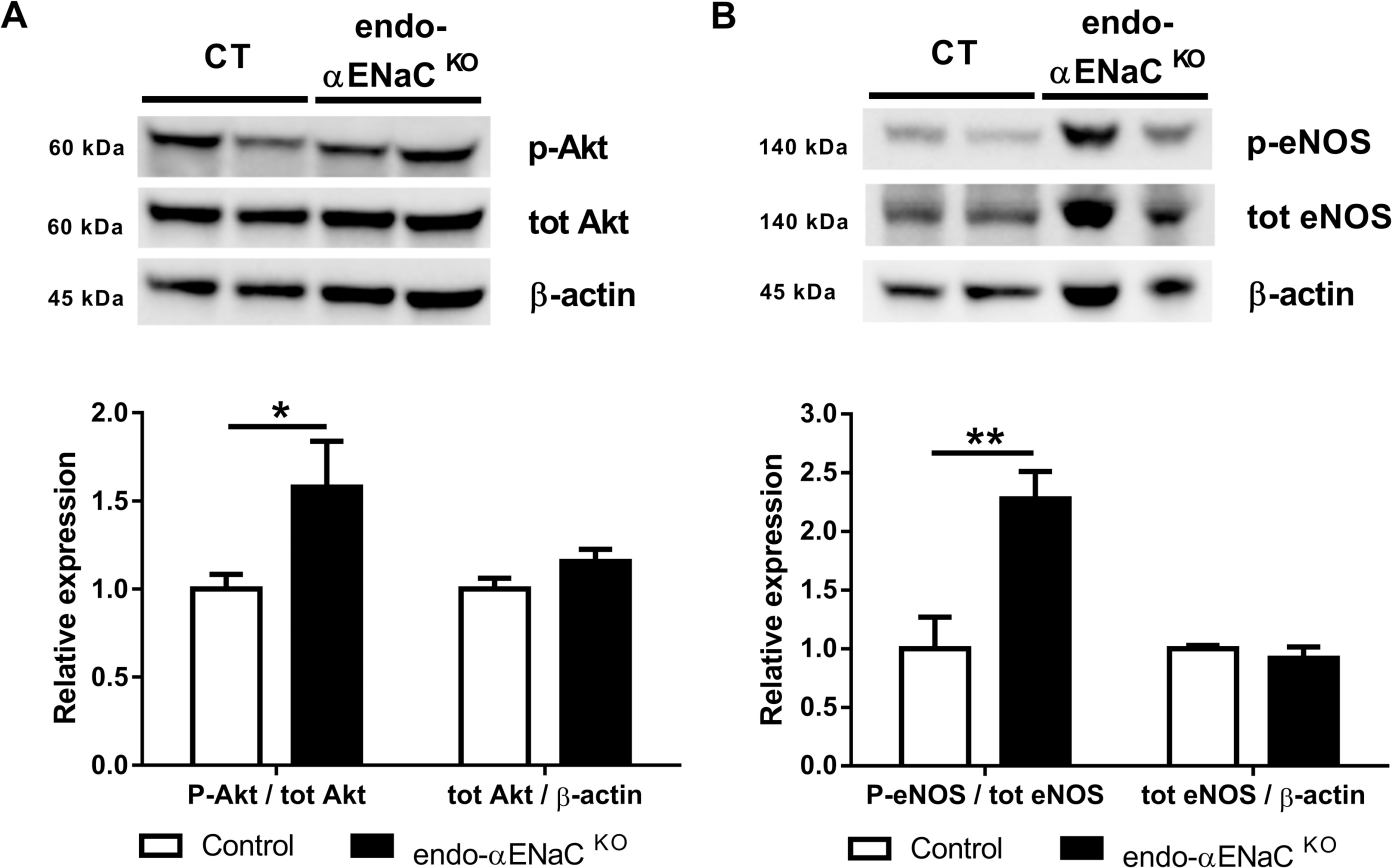
Consequences of αENaC gene inactivation on endothelial nitric oxide synthase activation pathway in the aorta. Western Blot analysis of protein expression of (A) phosphorylated Akt/total Akt ratio and total Akt/β-Actin ratio and (B) phosphorylated eNOS (S1177)/total eNOS ratio and total eNOS/β-Actin ratio in the aorta. White bars represent control mice and black bars represent endo-αENaC ^KO^ mice. Values are mean ± SEM (*n* = 9–10 for each group). *P<0.05, **P<0.01.

**Fig 5:**
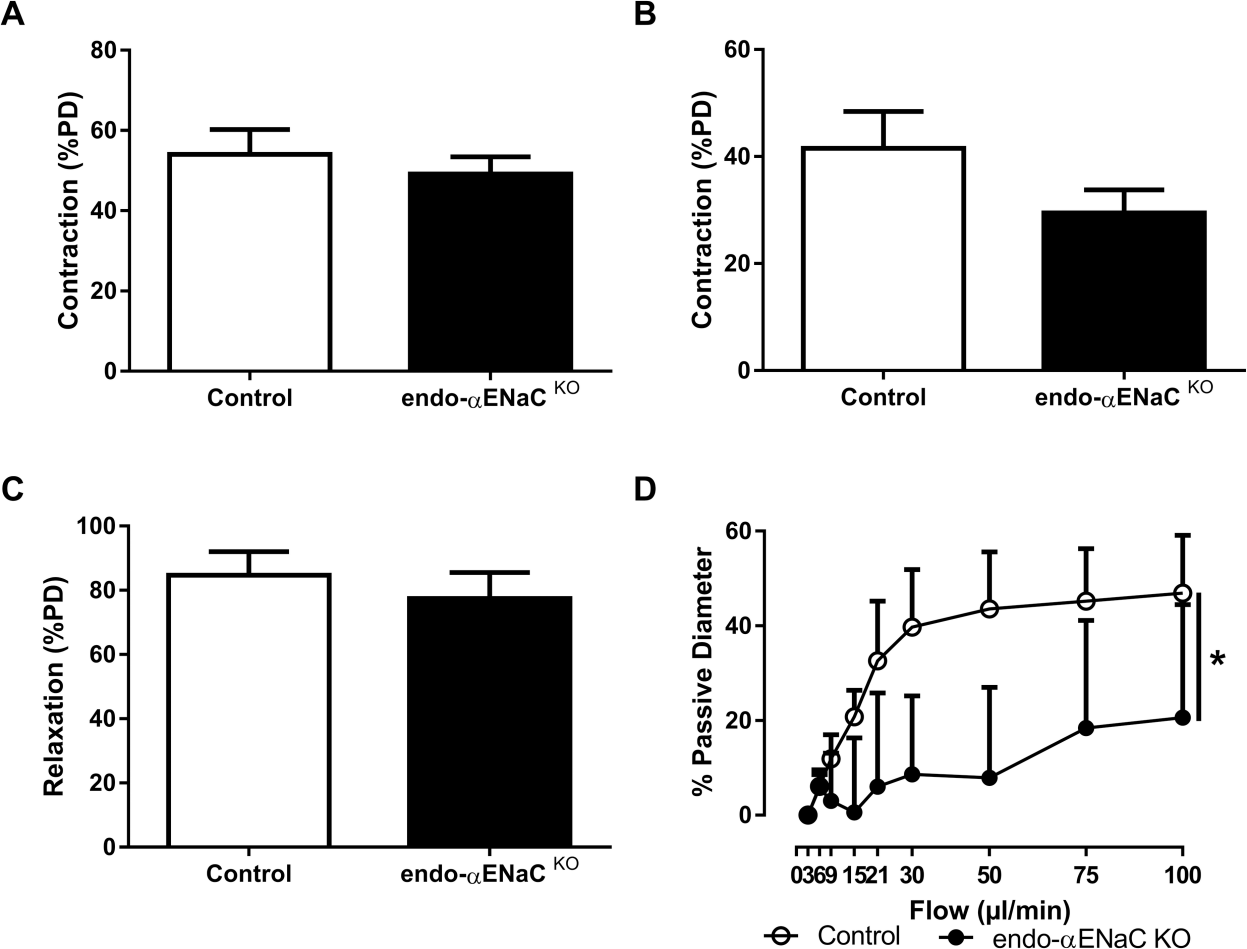
Effects of αENaC gene inactivation on vascular response in mesenteric arteries. Vasoactive responses in mesenteric arteries of endo-αENaC ^KO^ mice compared to their αENaC ^f/f^ control littermates. (A) Vasoconstrictive response to 8.10^−2^ M of potassium chloride. (B) Vasoconstrictive response to phenylephrine (C) Endothelium-dependent dilatory response induced by acetylcholine (D) Flow-mediated dilation in response to increased intraluminal flow. Values are mean ± SEM (*n* = 5–7 for each group). *P<0.05.

Endothelial NO is produced by activated endothelial nitric oxide synthase (eNOS) (by phosphorylation on the S1177 residue by phosphorylated Akt). In the aorta of endo-αENaC ^KO^ mice, steady state phosphorylated Akt and phosphorylated eNOS (S1177) levels were higher than in control mice (Fig 4A and 4B). This compensatory mechanism may explain similar vasoactive responses to potassium chloride (KCl), acetylcholine and phenylephrine observed in control and endo-αENaC ^KO^ vessels (Fig 5A–5C).

### Endothelial αENaC is involved in flow-mediated dilation

We next analyzed whether endothelial αENaC could be involved in vascular response to mechanical forces induced by blood flow. A plateau of dilation response was observed at 30 μl/min in littermate mice (Fig 5D) while the inactivation of the αENaC subunit in the endothelium almost fully inhibited the flow-mediated dilation in endo-αENaC ^KO^ (Fig 5D). This result highlights the importance of the endothelial αENaC subunit in the mechanotransduction of shear stress into vasodilation response.

## Discussion

Using a cell-specific knockout mouse model, we find two major implications of endothelial alpha subunit of the Epithelial Sodium Channel in vascular physiology. First, we show that the absence of αENaC expression induces to compensatory induction of phospho-eNOS and phospho-Akt, suggesting a crucial role of αENaC in basal condition. Second, we show that genetic deletion of the αENaC subunit in the endothelium leads to blunted flow-mediated dilation in resistant arteries. While the role of ENaC in shear stress sensing has been described in cultured human endothelial cells [8] we describe for the first time that endothelial αENaC modulates shear stress sensing *in vivo*.

The expression of the three α, β, γ subunits of ENaC has been reported in the endothelium of rat mesenteric arteries [6, 25]. The αENaC is expressed in human endothelial cells such as the ECV 304 cell line [5] and HUVEC [26]. Distinguishing the functional implication of the different subunits is not possible when using pharmacological ENaC blockers like amiloride or benzamil. A mouse model with endothelium-restricted αENaC subunit deletion allowed us to test the specific role of the αENaC subunit expressed in the endothelium. We observed 30% decreased in αENaC mRNA expression in whole aorta and no expression of αENaC protein at the surface of the endothelial cells. After mechanical removal of the endothelium, the aortas of the control group showed a decrease of more than 50% compared to control aortas with endothelium. Removal of the endothelium did not change expression in the endo-αENaC ^KO^ group (indicating that removal of endothelium did not change αENaC expression in the KO mice), so we cannot rule out that the remaining αENaC mRNA expression is related to non-endothelial cells. αENaC deletion did not alter the mRNA expression of the other subunits, βENaC and γENaC. While we were unable to study βENaC expression at the surface of endothelial cells, γENaC did not show any differences of expression between the endo-αENaC ^KO^ mice and their littermates. γENaC was expressed at the cell membrane in the absence of αENaC subunit. This was already observed in HEK-293 and COS-7 cells when γENaC was transfected in absence of αENaC [27]. Whether γENaC alone could have a role in vascular endothelium remains unknown.

Functional endothelial ENaC activity leads to amiloride-sensitive inward sodium current as reported in cultured endothelial cells [6, 7]. Liu *et al*. recently reported functional ENaC in intact endothelium of split-open rat mesenteric arteries [25]. One week of high salt diet decreased ENaC open probability in mesenteric arteries [25]. Decreased endothelial ENaC activity due to increased salt intake was associated with decreased eNOS phosphorylation [25]. Since reduced endothelial ENaC current induced by high salt diet was associated with an increased acetylcholine-mediated vasorelaxation, the decrease in eNOS S1177 phosphorylation may be a compensatory mechanism to prevent excessive relaxation [25]. Our results indicate that acute treatment with benzamil decreased acetylcholine-mediated NO production but that this effect was lost in endo-αENaC ^KO^ mice, which suggest an important role of endothelial αENaC in modulating agonist-mediated NO release in physiological conditions. of note, our western blot data were obtained in larger vessels (aorta), which might not be fully representative of mesenteric vessels, where the NO measurements where performed. Our data therefore points to 1) that endothelial αENaC subunit is involved for the pharmacological effect of benzamil in the vessel, 2) endothelial αENaC subunit is important for acetylcholine-induced NO production in mice, and 3) mice lacking endothelial αENaC subunit develop compensatory mechanisms to maintain NO production in response to acetylcholine. Strikingly, there was no compensatory mechanisms for flow-mediated dilation as compared to Ach-induced vasodilation.

The functional consequences of pharmacological inhibition of vascular ENaC on vasoreactivity have been analyzed *ex vivo* in rat mesenteric arteries and in mouse renal interlobar arteries. Perez *et al*. showed that acute treatment with amiloride or benzamil decreased contractile response to phenylephrine and serotonin [6] while Liu *et al*. showed increased acetylcholine-mediated vasodilation after amiloride pre-treatment [25]. In contrast, Jernigan *et al*. reported no effect of benzamil on phenylephrine-induced contraction in mouse interlobar arteries [28]. We show in the present study that constitutive endo-αENaC inactivation of endothelial αENaC in endo-αENaC ^KO^ mice does not alter contractile response, probably because compensatory mechanisms develop due to long-term absence of αENaC expression/activity in the endothelium. This suggests an important and non-redundant role of endothelial αENaC in endothelial physiology. ENaC activity in the endothelial cells have been shown to be regulated by some epoxyeicosatrienoic acids (EETs)[7] suggesting potential interaction with other vasoactive agents. Further studies should analyze the role of the other ENaC subunits in the function of endothelial ENaC.

In the present study, we confirmed an important role of endothelial αENaC in endothelial cortical cell stiffness: both pharmacological inhibition and targeted genetic inactivation of αENaC led to decreased stiffness of endothelial cells in *ex vivo* aorta preparations. Interestingly, we previously reported an opposite result in a mouse model with a β subunit mutation mimicking the Liddle mutation [29] (C-terminal truncation of the β subunit and increased endothelial surface expression of ENaC): increased endothelial ENaC surface expression led to an increase in endothelial cortical stiffness [15]. Acetylcholine-mediated NO release is preserved in endo-αENaC ^KO^ mice; however, the alteration of endothelial stiffness is maintained despite constitutive αENaC inactivation, which suggest that endothelial αENaC has a key role of in endothelial stiffness *in vivo*.

The Epithelial Na channel is expressed in the vasculature but its role in vascular function and organ damage is poorly understood. In this study, we showed that endothelial ENaC contributes to the regulation of agonist-induced NO production in the vasculature. Whether endothelial ENaC is involved in other pathologies associated with altered shear stress response, such as atherosclerosis or renal ischemia-reperfusion remains to be analyzed. The association of ENaC polymorphisms like the Trp493Ar or the T663A variants (which has been shown to decrease the functional and surface expression of hENaC in oocytes) and the increased risk of ischemic stroke, independently of blood pressure control, points to a possible link between αENaC and cardiovascular damage that needs to be further explored.

In conclusion, we demonstrate in this study that endothelial αENaC plays a critical role in vascular physiology, especially for shear-stress sensing and flow-mediated dilation. It would be important to now explore the implication of endothelial ENaC in human physiology and pathology.

## Supporting information

S1 Methods (DOCX)

S1 Fig Effect of mechanical removal of the endothelium on αENaC expression in the aorta. (A) Relative mRNA expression of the αENaC subunit in the aorta, with or without endothelium, of control mice (white bars) and endo-αENaC KO mice (black bars). Endo-αENaC KO mice present more than 50% decrease of αENaC expression compared to control in aortas with endothelium. After removal of the endothelium, αENaC expression in the control mice is decrease in the same proportion than in endo-αENaC KO mice. (B) Relative mRNA expression of von Willebrand Factor, a marker of endothelial cells. Endothelium removal decreases vWF expression around 75% in both groups, suggesting that some endothelial cells are still present after mechanical removal. (C) Relative mRNA expression of α-Smooth Muscle Actin, a marker of smooth muscle cells. Expression of αSMA is not altered by endothelium removal in neither of the groups. Values are mean ± SEM (n = 5 for each group).
(TIF)

S2 Fig Cre recombinase expression driven by the Tie2 promoter does not affect αENaC expression in the macrophages. Relative mRNA expression of the αENaC subunit in the macrophages, isolated from the peritoneal cavity or derived from the bone marrow, of control mice (white bars) and endo-αENaC ^KO^ mice (black bars). Values are mean ± SEM (*n* = 5 for each group).
(TIF)

S3 Fig Genetic deletion of endothelial αENaC subunit decrease αENaC protein expression on the surface of endothelial cells, but not γENaC. Representative images of quantum dot (QD)-immunostaining for αENaC and γENaC on the surface of control and endo-αENaC ^KO^ aortic endothelial cells. Scale bar represents 30 μm.
(TIF)

S4 Fig Inactivation of endothelial αENaC subunit has no consequences on renal sodium handling. Ratio of urinary sodium (U Na^+^) on urinary creatinine (U Creat) following (A) acute sodium load, (B) acute amiloride injection or (C) 4 days of low salt diet (0.1% NaCl). White bars represent control mice and black bars represent endo-αENaC ^KO^ mice. Values are mean ± SEM (*n* = 10 for each group).
(TIF)

S1 Table Primer sequences. (DOCX)

S2 Table Antibodies and dilutions used in western blot. (DOCX)

S3 Table Characterization of the vessel structure of the endo-αENaC ^KO^ mouse model. (DOCX)
