# Supplementary material for: The endothelial αENaC contributes to vascular endothelial function in vivo
Source: PLoS One. 2017 Sep 26;12(9):e0185319. doi: 10.1371/journal.pone.0185319 (PMC5614594; doi:10.1371/journal.pone.0185319)
Supplement: S1 Methods — (DOCX) [file pone.0185319.s001.docx]

**Supplementary methods**

**In situ organ fixation and histological analyses**

Animals were anesthetized with a mix of ketamine/xylazine. Blood was flushed with PBS at 37°C followed by perfusion at physiological pressure (100mmHg) of a paraformaldehyde 4% (PFA) solution at 37°C for 30 min. Organs were collected and placed in PFA for 24 hours. Vessels were then rinsed and embedded in paraffin. 4 μm sections of thoracic aortas, carotids and 1^st^ order mesenteric arteries were stained with Orcein for elastin content and morphological analysis. Image acquisition was made using a Leica DM4000B microscope and Leica Application Suite software (Leica, Germany). All Images were captured at the same depth and with identical settings for laser, gain and offset intensity. Wall thickness and diameter were measured in two different regions in duplicate. All measurements were performed blind using ImageJ software. Images were calibrated with known standards.

**Vascular Reactivity Analysis**

Vascular contractile and relaxing responses in isolated thoracic aortas were assessed as previously described ([1](#_ENREF_1)). Aortic rings were maximally contracted with isotonic potassium chloride solution (80mM) and then contractile capacity of each ring was measured with a submaximal concentration of phenylephrine (10^-5^M). The contraction evoked by phenylephrine (10^-9^ to 10^-5^M) tested the α-adrenergic pathway. The dose response to acetylcholine (10^-9^ to 10^-6^) was determined in rings precontracted by 10^-5^M phenylephrine to evaluate endothelium dependent relaxation. All the chemicals were purchase from Sigma-Aldrich if not specified otherwise.

Mesenteric arteries were cannulated at both ends in a video-monitored perfusion system (LSI) as previously described ([2](#_ENREF_2)). Briefly, arteries were bathed in a PSS of the following composition (mM): 130, NaCl; 15, NaHCO_3_; 3.7, KCl; 1.2 KH_2_PO_4_; 1.2, MgSO_4_; 11, glucose; 1.6, CaCl_2_; and 5, HEPES, pH 7.4, PO_2_ 160 mmHg, PCO_2_ 37 mmHg) and pressure was maintained at 75mmHg by a servoperfusion system. After 20 minutes arterial segments reactivity was estimated using first a potassium rich solution (80 mM). After that, endothelial function was evaluated using acetylcholine (10^-6^ M) after precontraction with phenylephrine (10^-6^ M). At the end of each experiment, arterial segments were perfused with a Ca^2+^-free physiological salt solution containing ethylenbis-(oxyethylenenitrolo) tetra-acetic acid (EGTA; 2 mM), papaverin (10^-4^M) and SNP (10^-5^M); then passive diameter of the arteries was measured at 75mmHg ([3](#_ENREF_3)). Results were expressed as the percentage of passive diameter (measured diameter/passive diameter×100).

**Mechanical removal of the endothelium**

Thoracic aorta was obtained from endo-αENaC KO or control littermate mice and was quickly cleaned from the surrounding tissues under microscope. Half of the aorta was directly frozen in liquid nitrogen. The second half was submitted to endothelium removal. The endothelial cells were removed mechanically with the insertion of a wire inside the lumen of the aorta. After that, the aorta was open longitudinally and gently scrapped with a cell scraper and frozen until RNA extraction.

**Isolation of macrophages**

Bone marrow derived macrophages (BMDMs) were isolated from male endo-αENaC KO or control littermate mice by flushing the femur and tibia with PBS. The bone marrow cells were resuspended in RPMI-1640 containing 1% L-glutamine, 1% Pen/Strep, 1% pyruvate and 10% FBS and 100 ng/mL M-CSF. Cells were incubated for 10 days at 37 °C and 5% CO2 with medium change every 3–4 day. Peritoneal macrophages were isolated from male endo-αENaC KO or control littermate mice. Peritoneal membrane was separated from under the abdominal musculature. 5-7 ml ice cold PBS was injected into peritoneal cavity; peritoneum was gently and completely massaged; PBS was then aspirated from peritoneal cavity. Peritoneal cells were resuspended in RPMI-1640 containing 1% L-glutamine, 1% Pen/Strep and 10% FBS. Cells were incubated for 24 hours at 37 °C and 5% CO2.

1. Galmiche G, Labat C, Mericskay M, Aissa KA, Blanc J, Retailleau K, et al. Inactivation of serum response factor contributes to decrease vascular muscular tone and arterial stiffness in mice. Circulation research. 2013;112(7):1035-45.

2. Loufrani L, Levy BI, Henrion D. Defect in microvascular adaptation to chronic changes in blood flow in mice lacking the gene encoding for dystrophin. Circulation research. 2002;91(12):1183-9.

3. Henrion D, Terzi F, Matrougui K, Duriez M, Boulanger CM, Colucci-Guyon E, et al. Impaired flow-induced dilation in mesenteric resistance arteries from mice lacking vimentin. J Clin Invest. 1997;100(11):2909-14.

**Supplementary tables**

**S1 Table: Primer sequences**

|  |  |  |  |  |  |  |
| --- | --- | --- | --- | --- | --- | --- |
|  |  |  |  |  |  |  |
| **Primers** | **Forward** | | | **Reverse** | | |
|  |  |  |  |  |  |  |
|  |  |  |  |  |  |  |
| 18S (*Rn18s,* MGI:97943) | CGC CGC TAG AGG TGA AAT TC | | | TCT TGG CAA ATG CTT TCG C | | |
| αENaC (*Scnn1a,* MGI:101782) | CGG AGT TGC TAA ACT CAA CAT C | | | TGG AGA CCA GTA CCG GC T | | |
| βENaC (*Scnn1b,* MGI:104696) | GAC TTC CCA GAC TGG GCC TAT | | | GGT CAC ACT CAT CTG CAG GTT TAG | | |
| γENaC (*Scnn1g,* MGI:104695) | GCA AGC AAT CCT GCA GCT TT | | | GTG CCA AGC TGG TGG TCA GT | | |
| Renin (*Ren1,* MGI:97898) | GCC GCC TCT ACC TTG CTT GTG | | | GGG GCA GCT CGG TGA CCT CT | | |

ENaC, epithelial sodium channel.

**S2 Table: Antibodies and dilutions used in western blots**

|  |  |  |  |  |  |  |
| --- | --- | --- | --- | --- | --- | --- |
|  | **Company** | | | **Dilution** | | |
|  |  |  |  |  |  |  |
|  |  |  |  |  |  |  |
| ***Primary antibodies*** |  | | |  | | |
| Anti-Akt (phospho Ser^473^) | Cell Signaling (9271) | | | 1/1000 BSA | | |
| Anti-Akt | Cell Signaling (9272) | | | 1/1000 BSA | | |
| Anti-eNOS (phospho Ser^1177^) | Abcam (ab75639) | | | 1/1000 milk | | |
| Anti-eNOS | Santa Cruz (sc654) | | | 1/200 milk | | |
| Anti-β-actin | Abcam (ab8227) | | | 1/1000 milk | | |
|  |  | | |  | | |
| ***Secondary antibodies*** |  | | |  | | |
| Anti-Rabbit IgG | GE healthcare (na934) | | | 1/10000 milk | | |

BSA, bovine serum albumin; eNOS, endothelial nitric oxide synthase

**S3 Table: Characterization of the vessel structure of the** **endo-αENaC ^KO^ mouse model*.***

|  | **Control (n=11)** | | | **Endo-αENaC ^KO^ (n=9)** | | |
| --- | --- | --- | --- | --- | --- | --- |
|  |  |  |  |  |  |  |
|  |  |  |  |  |  |  |
| ***Thoracic Aorta*** 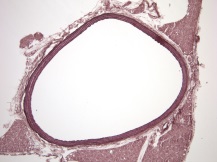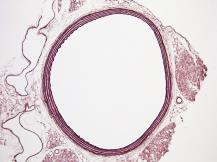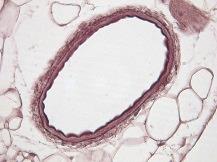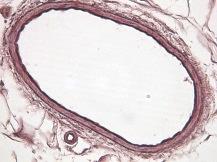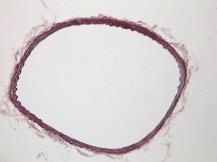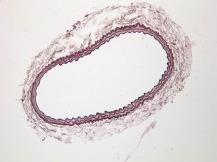 |  |  |  |  |  |  |
| Wall Thickness (µm) | 36.8 | ± | 1.1 | 38.2 | ± | 3.2 |
| Diameter (µm) | 790.0 | ± | 30.6 | 782.2 | ± | 33.1 |
| ***Carotide*** |  |  |  |  |  |  |
| Wall Thickness (µm) | 15.1 | ± | 0.9 | 25.3 | ± | 6.6 |
| Diameter (µm) | 327.6 | ± | 11.0 | 282.4 | ± | 52.7 |
| ***Mesenteric arteries (1^st^ order)*** |  |  |  |  |  |  |
| Wall Thickness (µm) | 8.4 | ± | 0.8 | 10.2 | ± | 1.7 |
| Diameter (µm) | 145.3 | ± | 27.0 | 107.7 | ± | 14.1 |

**Supplementary figures**


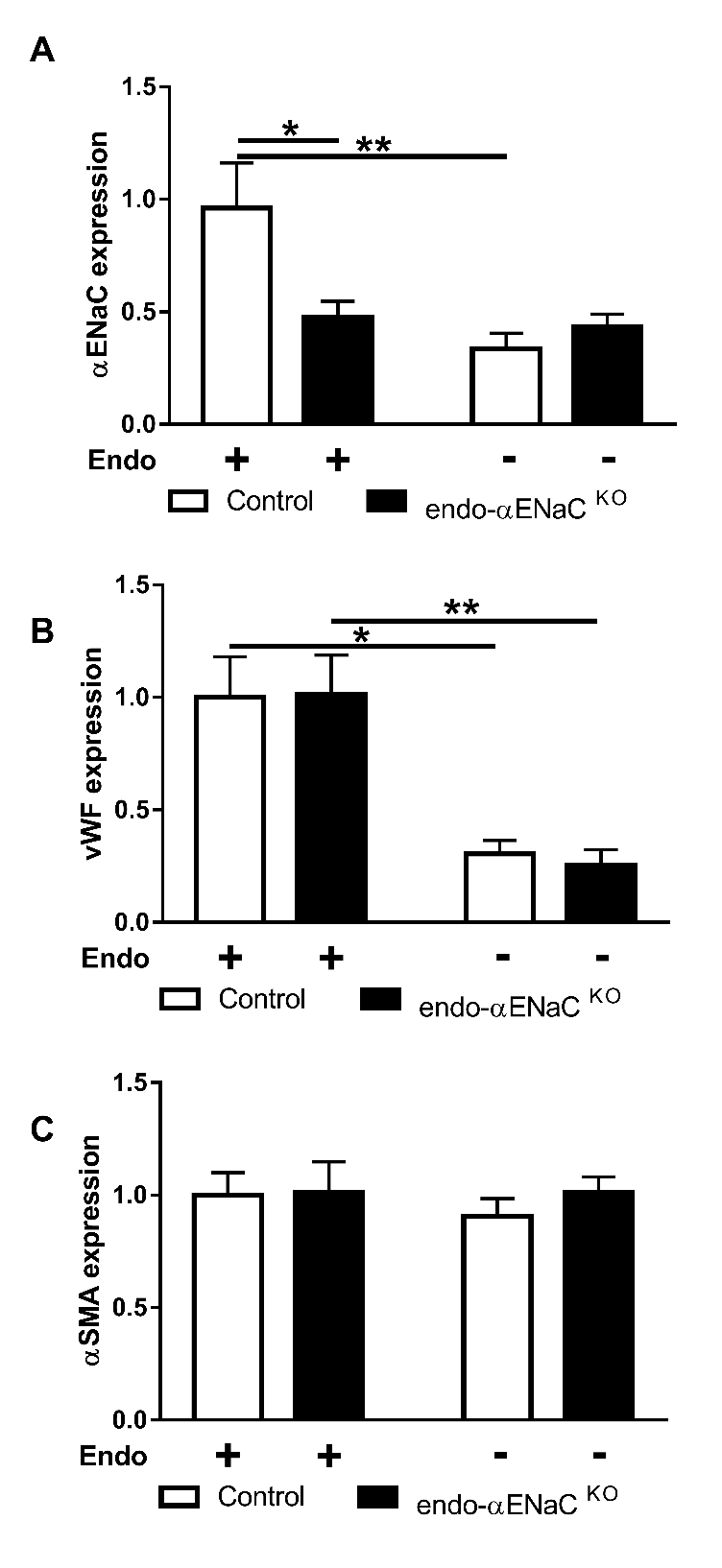


**S1 Fig: Effect of mechanical removal of the endothelium on αENaC expression in the aorta.** (A) Relative mRNA expression of the αENaC subunit in the aorta, with or without endothelium, of control mice (white bars) and endo-αENaC ^KO^ mice (black bars). Endo-αENaC ^KO^ mice present more than 50% decrease of αENaC expression compared to control in aortas with endothelium. After removal of the endothelium, αENaC expression in the control mice is decrease in the same proportion than in endo-αENaC ^KO^ mice. (B) Relative mRNA expression of von Willebrand Factor, a marker of endothelial cells. Endothelium removal decreases vWF expression around 75% in both groups, suggesting that some endothelial cells are still present after mechanical removal. (C) Relative mRNA expression of α-Smooth Muscle Actin, a marker of smooth muscle cells. Expression of αSMA is not altered by endothelium removal in neither of the groups. Values are mean ± SEM (*n*=5 for each group).

**
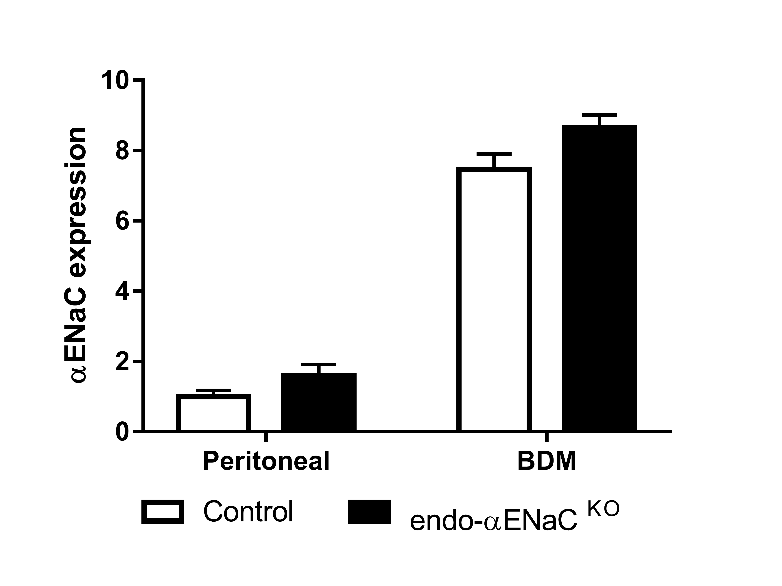
**

**S2 Fig: Cre recombinase expression driven by the Tie2 promoter does not affect αENaC expression in the macrophages.** Relative mRNA expression of the αENaC subunit in the macrophages, isolated from the peritoneal cavity or derived from the bone marrow, of control mice (white bars) and endo-αENaC ^KO^ mice (black bars). Values are mean ± SEM (*n*=5 for each group).

**
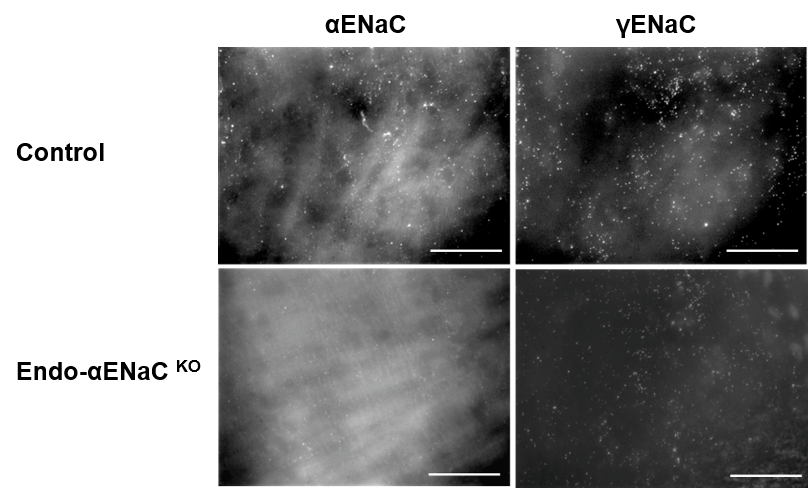
**

**S3 Fig: Genetic deletion of endothelial αENaC subunit decrease αENaC protein expression on the surface of endothelial cells, but not γENaC.** Representative images of quantum dot (QD)-immunostaining for αENaC and γENaC on the surface of control and endo-αENaC ^KO^ aortic endothelial cells. Scale bar represents 30 µm.

**
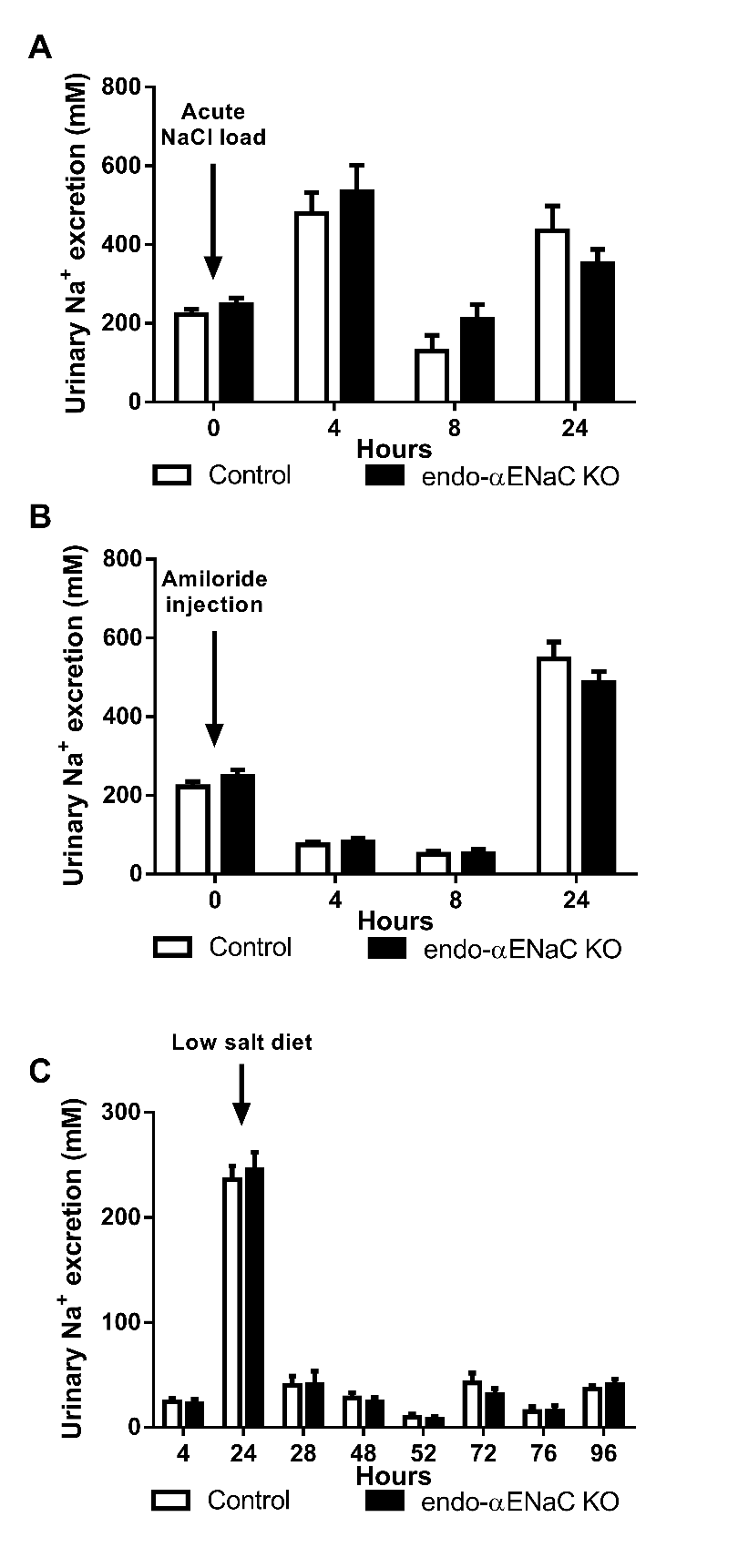
**

**S4 Fig: Inactivation of endothelial αENaC subunit has no consequences on renal sodium handling.** Ratio of urinary sodium (U Na^+^) on urinary creatinine (U Creat) following (A) acute sodium load, (B) acute amiloride injection or (C) 4 days of low salt diet (0.1 % NaCl). White bars represent control mice and black bars represent endo-αENaC ^KO^ mice. Values are mean ± SEM (*n*=10 for each group).
