# Supplementary figures and images for: The endothelial αENaC contributes to vascular endothelial function in vivo
Source: PLoS One. 2017 Sep 26;12(9):e0185319. doi: 10.1371/journal.pone.0185319 (PMC5614594; doi:10.1371/journal.pone.0185319)

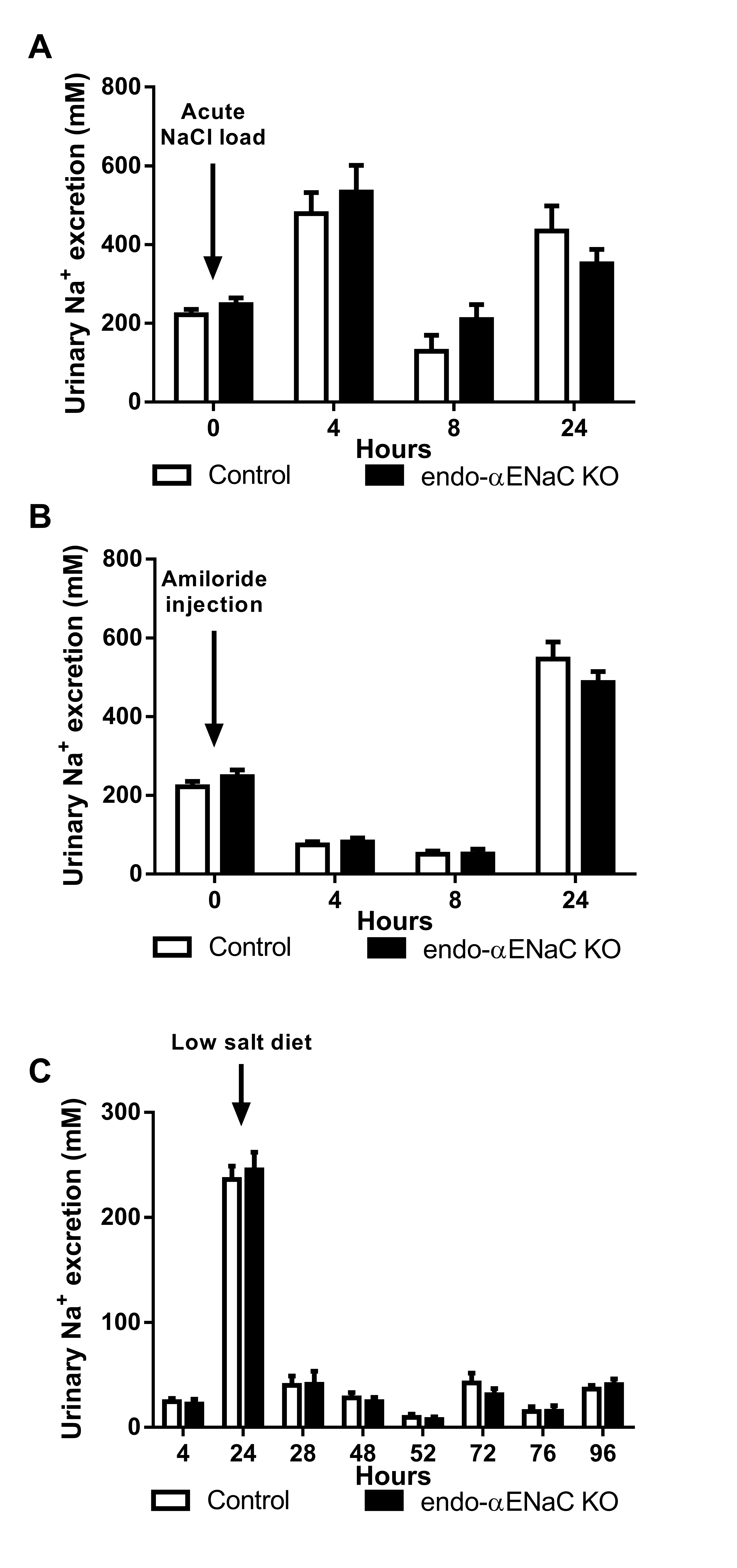

Supplement: S1 Fig — (A) Relative mRNA expression of the αENaC subunit in the aorta, with or without endothelium, of control mice (white bars) and endo-αENaC KO mice (black bars). Endo-αENaC KO mice present more than 50% decrease of αENaC expression compared to control in aortas with endothelium. After removal of the endothelium, αENaC expression in the control mice is decrease in the same proportion than in endo-αENaC KO mice. (B) Relative mRNA expression of von Willebrand Factor, a marker of endothelial cells. Endothelium removal decreases vWF expression around 75% in both groups, suggesting that some endothelial cells are still present after mechanical removal. (C) Relative mRNA expression of α-Smooth Muscle Actin, a marker of smooth muscle cells. Expression of αSMA is not altered by endothelium removal in neither of the groups. Values are mean ± SEM (n = 5 for each group). (TIF) [file pone.0185319.s002.tif]

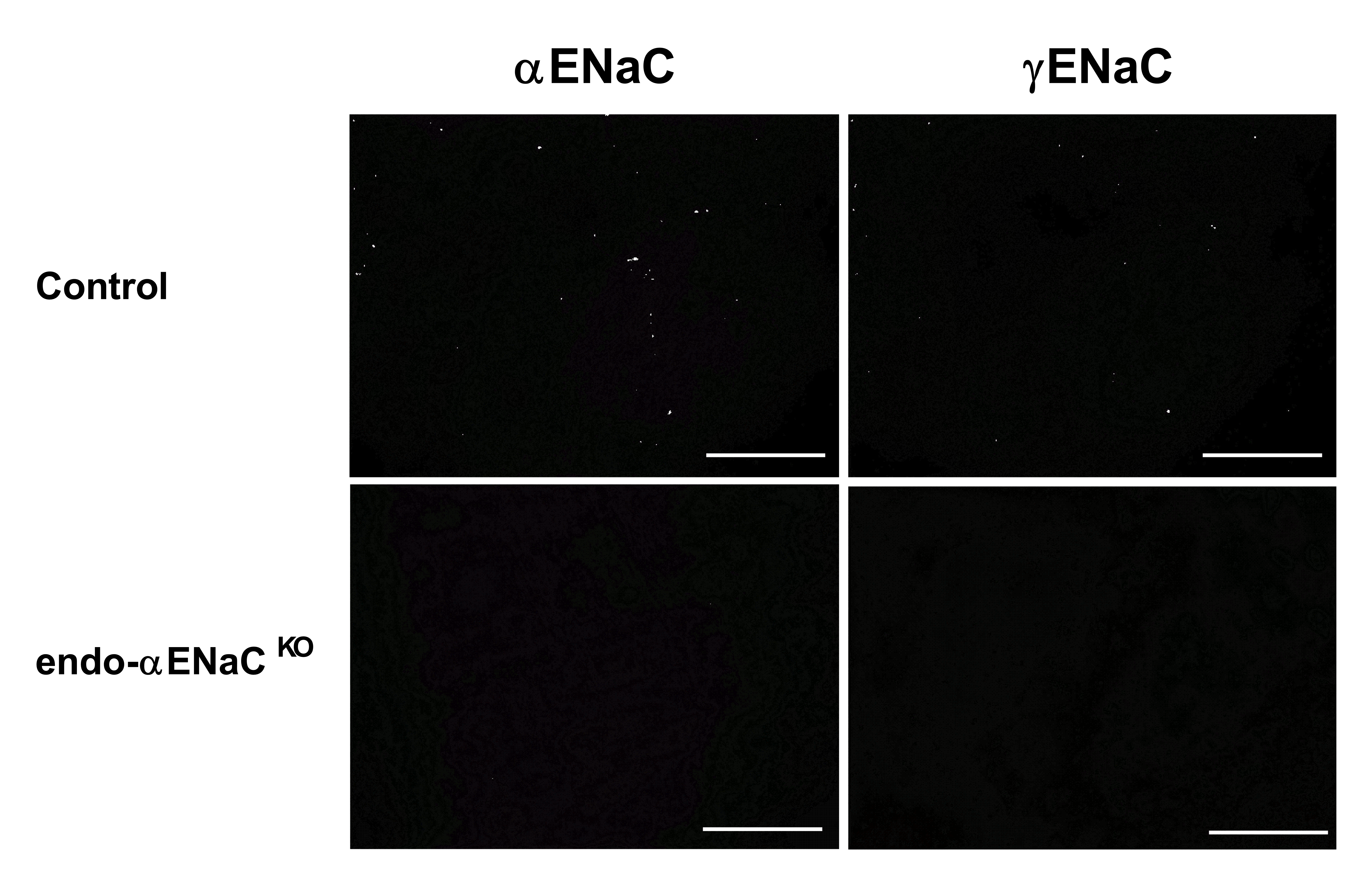

Supplement: S2 Fig — Relative mRNA expression of the αENaC subunit in the macrophages, isolated from the peritoneal cavity or derived from the bone marrow, of control mice (white bars) and endo-αENaC KO mice (black bars). Values are mean ± SEM (n = 5 for each group). (TIF) [file pone.0185319.s003.tif]

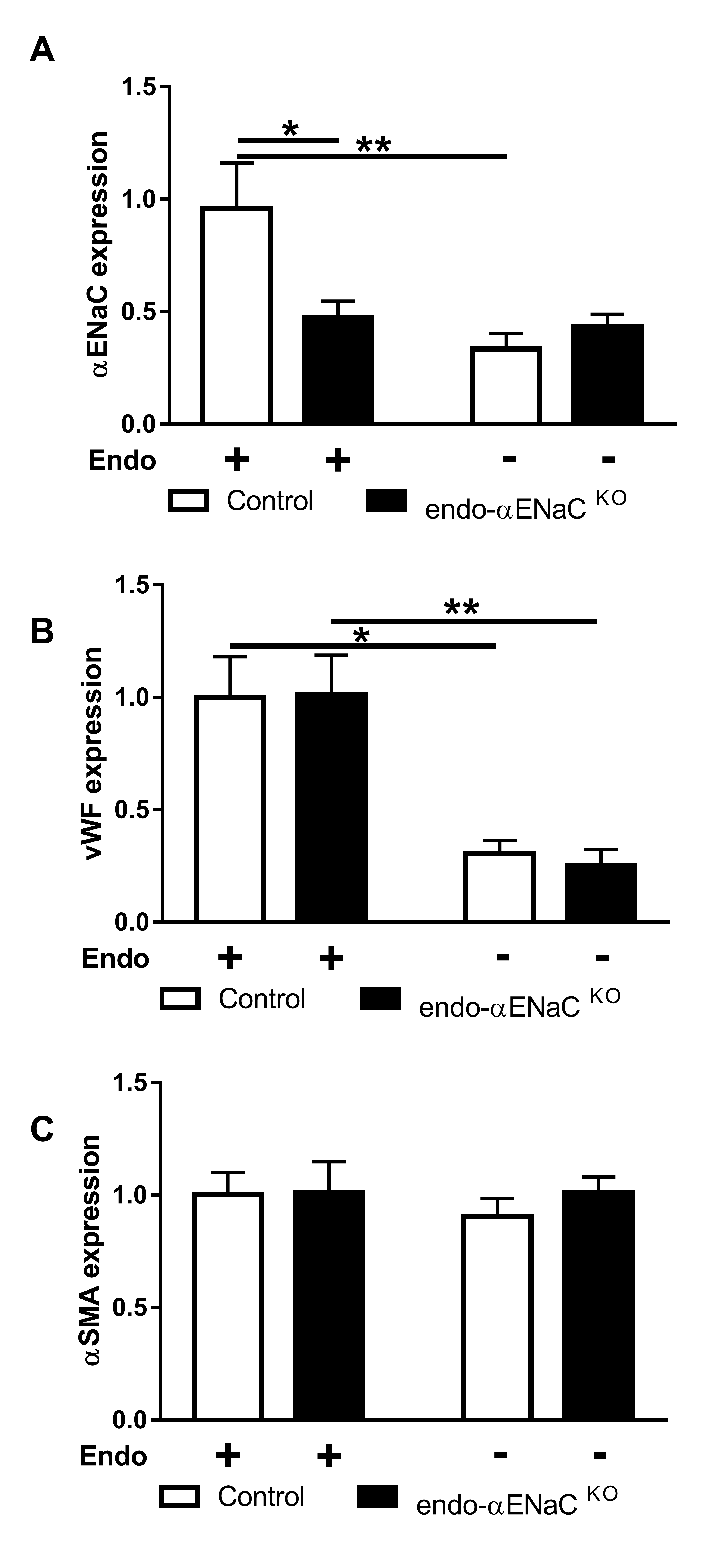

Supplement: S3 Fig — Representative images of quantum dot (QD)-immunostaining for αENaC and γENaC on the surface of control and endo-αENaC KO aortic endothelial cells. Scale bar represents 30 μm. (TIF) [file pone.0185319.s004.tif]

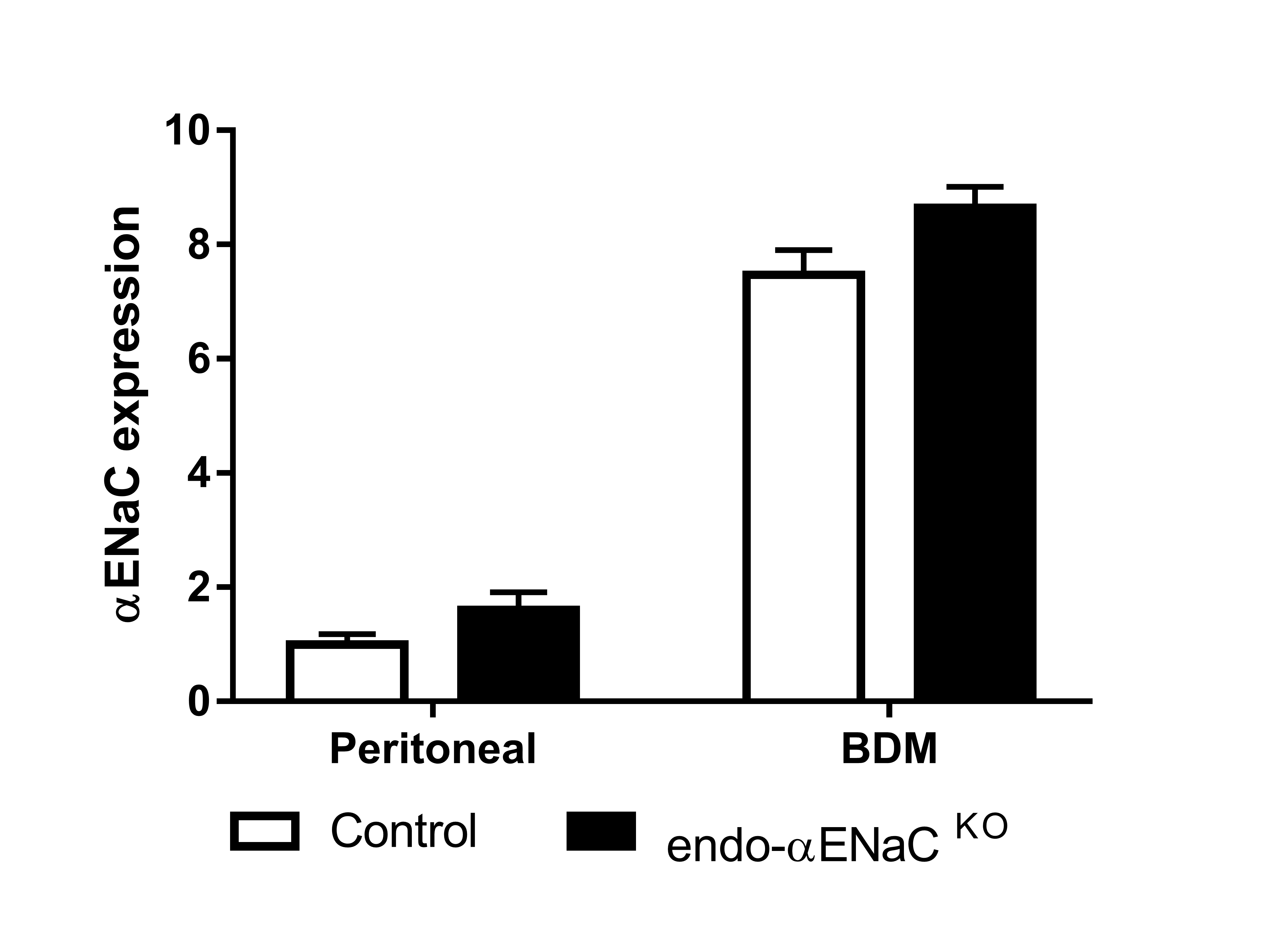

Supplement: S4 Fig — Ratio of urinary sodium (U Na+) on urinary creatinine (U Creat) following (A) acute sodium load, (B) acute amiloride injection or (C) 4 days of low salt diet (0.1% NaCl). White bars represent control mice and black bars represent endo-αENaC KO mice. Values are mean ± SEM (n = 10 for each group). (TIF) [file pone.0185319.s005.tif]
