# Supplementary material for: The endothelial αENaC contributes to vascular endothelial function in vivo
Source: PLoS One. 2017 Sep 26;12(9):e0185319. doi: 10.1371/journal.pone.0185319 (PMC5614594; doi:10.1371/journal.pone.0185319)
Supplement: S1 Table — (DOCX) [file pone.0185319.s006.docx]

**S1 Table: Primer sequences**

|  |  |  |  |  |  |  |
| --- | --- | --- | --- | --- | --- | --- |
|  |  |  |  |  |  |  |
| **Primers** | **Forward** | | | **Reverse** | | |
|  |  |  |  |  |  |  |
|  |  |  |  |  |  |  |
| 18S (*Rn18s,* MGI:97943) | CGC CGC TAG AGG TGA AAT TC | | | TCT TGG CAA ATG CTT TCG C | | |
| αENaC (*Scnn1a,* MGI:101782) | CGG AGT TGC TAA ACT CAA CAT C | | | TGG AGA CCA GTA CCG GC T | | |
| βENaC (*Scnn1b,* MGI:104696) | GAC TTC CCA GAC TGG GCC TAT | | | GGT CAC ACT CAT CTG CAG GTT TAG | | |
| γENaC (*Scnn1g,* MGI:104695) | GCA AGC AAT CCT GCA GCT TT | | | GTG CCA AGC TGG TGG TCA GT | | |
| Renin (*Ren1,* MGI:97898) | GCC GCC TCT ACC TTG CTT GTG | | | GGG GCA GCT CGG TGA CCT CT | | |

ENaC, epithelial sodium channel.
