# Supplementary material for: The endothelial αENaC contributes to vascular endothelial function in vivo
Source: PLoS One. 2017 Sep 26;12(9):e0185319. doi: 10.1371/journal.pone.0185319 (PMC5614594; doi:10.1371/journal.pone.0185319)
Supplement: S2 Table — (DOCX) [file pone.0185319.s007.docx]

**S2 Table: Antibodies and dilutions used in western blots**

|  |  |  |  |  |  |  |
| --- | --- | --- | --- | --- | --- | --- |
|  | **Company** | | | **Dilution** | | |
|  |  |  |  |  |  |  |
|  |  |  |  |  |  |  |
| ***Primary antibodies*** |  | | |  | | |
| Anti-Akt (phospho Ser^473^) | Cell Signaling (9271) | | | 1/1000 BSA | | |
| Anti-Akt | Cell Signaling (9272) | | | 1/1000 BSA | | |
| Anti-eNOS (phospho Ser^1177^) | Abcam (ab75639) | | | 1/1000 milk | | |
| Anti-eNOS | Santa Cruz (sc654) | | | 1/200 milk | | |
| Anti-β-actin | Abcam (ab8227) | | | 1/1000 milk | | |
|  |  | | |  | | |
| ***Secondary antibodies*** |  | | |  | | |
| Anti-Rabbit IgG | GE healthcare (na934) | | | 1/10000 milk | | |

BSA, bovine serum albumin; eNOS, endothelial nitric oxide synthase
