# Supplementary material for: The endothelial αENaC contributes to vascular endothelial function in vivo
Source: PLoS One. 2017 Sep 26;12(9):e0185319. doi: 10.1371/journal.pone.0185319 (PMC5614594; doi:10.1371/journal.pone.0185319)
Supplement: S3 Table — (DOCX) [file pone.0185319.s008.docx]

**S3 Table: Characterization of the vessel structure of the** **endo-αENaC ^KO^ mouse model*.***

|  | **Control (n=11)** | | | **Endo-αENaC ^KO^ (n=9)** | | |
| --- | --- | --- | --- | --- | --- | --- |
|  |  |  |  |  |  |  |
|  |  |  |  |  |  |  |
| ***Thoracic Aorta*** 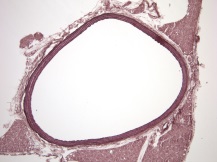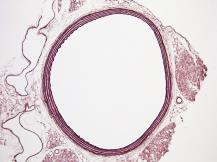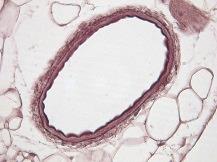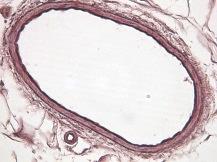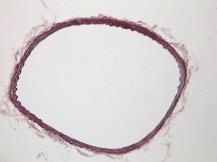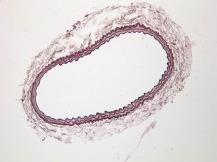 |  |  |  |  |  |  |
| Wall Thickness (µm) | 36.8 | ± | 1.1 | 38.2 | ± | 3.2 |
| Diameter (µm) | 790.0 | ± | 30.6 | 782.2 | ± | 33.1 |
| ***Carotide*** |  |  |  |  |  |  |
| Wall Thickness (µm) | 15.1 | ± | 0.9 | 25.3 | ± | 6.6 |
| Diameter (µm) | 327.6 | ± | 11.0 | 282.4 | ± | 52.7 |
| ***Mesenteric arteries (1^st^ order)*** |  |  |  |  |  |  |
| Wall Thickness (µm) | 8.4 | ± | 0.8 | 10.2 | ± | 1.7 |
| Diameter (µm) | 145.3 | ± | 27.0 | 107.7 | ± | 14.1 |
